# Multiple losses of photosynthesis and convergent reductive genome evolution in the colourless green algae *Prototheca*

**DOI:** 10.1038/s41598-017-18378-8

**Published:** 2018-01-17

**Authors:** Shigekatsu Suzuki, Rikiya Endoh, Ri-ichiroh Manabe, Moriya Ohkuma, Yoshihisa Hirakawa

**Affiliations:** 1 Graduate School of Life and Environmental Sciences, University of Tsukuba, Ibaraki, Japan; 2 Center for Environmental Biology and Ecosystem Studies, National Institute of Environmental Studies, Ibaraki, Japan; 3 Japan Collection of Microorganisms, RIKEN BioResource Center, Ibaraki, Japan; 4 Division of Genomic Technologies, RIKEN Center for Life Science Technologies, Kanagawa, Japan; 5 Faculty of Life and Environmental Sciences, University of Tsukuba, Ibaraki, Japan

## Abstract

Autotrophic eukaryotes have evolved by the endosymbiotic uptake of photosynthetic organisms. Interestingly, many algae and plants have secondarily lost the photosynthetic activity despite its great advantages. *Prototheca* and *Helicosporidium* are non-photosynthetic green algae possessing colourless plastids. The plastid genomes of *Prototheca wickerhamii* and *Helicosporidium* sp. are highly reduced owing to the elimination of genes related to photosynthesis. To gain further insight into the reductive genome evolution during the shift from a photosynthetic to a heterotrophic lifestyle, we sequenced the plastid and nuclear genomes of two *Prototheca* species, *P. cutis* JCM 15793 and *P. stagnora* JCM 9641, and performed comparative genome analyses among trebouxiophytes. Our phylogenetic analyses using plastid- and nucleus-encoded proteins strongly suggest that independent losses of photosynthesis have occurred at least three times in the clade of *Prototheca* and *Helicosporidium*. Conserved gene content among these non-photosynthetic lineages suggests that the plastid and nuclear genomes have convergently eliminated a similar set of photosynthesis-related genes. Other than the photosynthetic genes, significant gene loss and gain were not observed in *Prototheca* compared to its closest photosynthetic relative *Auxenochlorella*. Although it remains unclear why loss of photosynthesis occurred in *Prototheca*, the mixotrophic capability of trebouxiophytes likely made it possible to eliminate photosynthesis.

## Introduction

Acquisition of photosynthesis occurred in diverse eukaryotes by several endosymbiotic events wherein a photosynthetic organism was engulfed and integrated into a heterotrophic protist^1,2^. Phototrophic organisms can generate reduced carbon compounds in their plastids via the conversion of freely available light energy. Despite the great advantages, loss of photosynthesis has occurred in diverse lineages of organisms (e.g. apicomplexans, chlorophytes, cryptophytes, diatoms, dinoflagellates, euglenophytes, and Orobanchaceae species), along with heterotrophic free-living algae, holoparasitic plants, and pathogenic protists^3^. Such non-photosynthetic organisms survive by the uptake of organic carbon from the environment or host cells.

During the process of photosynthesis loss, plastids are generally reduced with regards to function, structure, and genome. Plastid genomes of non-photosynthetic organisms, except for *Polytoma uvella*^4^, are commonly smaller in size than that of the photosynthetic plastid genomes, because of the loss of genes related to photosynthesis, such as photochemical reaction complexes^5^. Particularly, the free-living green algae *Polytomella*^6^, the holoparasitic plant *Rafflesia lagascae*^7^, and the pathogenic alveolate *Perkinsus marinus*^8^ lack whole plastid genomes. Non-photosynthetic plastids lack the ability for light harvesting, photochemical reactions, and chlorophyll biosynthesis, whereas a part of the photosynthesis-related biosynthesis pathways is often retained. It has been reported that the nuclear genome of non-photosynthetic plastid-bearing organisms still encodes proteins for several plastid metabolic pathways, such as carbon fixation, fatty acid, terpenoid, tetrapyrrole, and isoprenoid biosynthesis^9,10^. Therefore, colourless plastids still possess some important functions other than those involved in photosynthesis.

Trebouxiophyte green algae include two non-photosynthetic genera, *Prototheca* and *Helicosporidium*, which are closely related to the photosynthetic genera, *Chlorella* and *Auxenochlorella*^11–13^. The genus *Prototheca* consists of free-living heterotrophic species, which exist in the soil and aqueous environments as ubiquitous organisms, and sometimes cause infections, termed protothecosis in animals, including humans^14,15^. The genus *Helicosporidium* is known to infect a variety of invertebrates; and *in vitro* axenic cultures are available for some strains^16^. Both *Prototheca* and *Helicosporidium* are believed to possess colourless plastids because of the presence of plastid genomes. Ultrastructural studies showed that *Prototheca* cells have a plastid-like structure surrounded by two membranes and filled by starch granules^17,18^. To date, complete plastid genomes of *Prototheca wickerhamii* and *Helicosporidium* sp. ATCC50920 have been reported^19,20^. The respective genomes encode 40 and 26 proteins, and lack most of the photosynthesis-related genes, though the plastid genome of *P. wickerhamii* contains six genes for ATP synthase. A comparative analysis revealed that the gene order excluding the absent genes is highly conserved in *P. wickerhamii* and its closest known photosynthetic relative *Auxenochlorella protothecoides*^19^. The plastid genome of *Helicosporidium* sp. is the smallest among the available plastid genomes of green algae^20^, and its gene order is diversified compared to *Prototheca*^19^. The nuclear genome of *Helicosporidium* sp. has been sequenced^21^, which revealed that many nuclear genes for the light-harvesting complexes, photosystems, and pigment biosynthesis have been lost; whereas part of photosynthesis-related functions, such as carbon fixation and terpenoid biosynthesis, have been retained.

To gain further insight into the genome evolution during the shift from a photosynthetic to a heterotrophic lifestyle in trebouxiophytes, we sequenced the plastid and nuclear genomes of two *Prototheca* species, *P. cutis* (JCM 15793 strain) and *P. stagnora* (JCM 9641 strain). Our phylogenetic analyses using plastid- and nucleus-encoded proteins strongly suggest that independent losses of photosynthesis have occurred at least three times in *Prototheca* and *Helicosporidium*. Comparative analyses of the plastid and nuclear genomes revealed that the gene content for plastid functions was highly conserved among the non-photosynthetic lineages, and the photosynthesis-related genes have mostly disappeared. Our findings suggest that non-photosynthetic trebouxiophytes have convergently lost a similar set of genes related to photosynthesis.

## Results and Discussion

### Overview of plastid and nuclear genomes of *P. cutis* and *P. stagnora*

**Figure 1:**
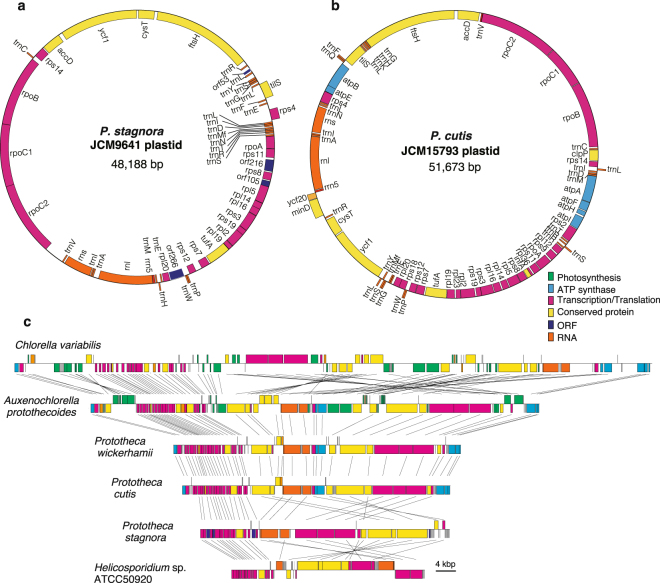
Structure of the plastid genomes of *P. stagnora* and *P. cutis*. (**a**,**b**) Gene maps of the plastid genomes of *P. stagnora* and *P. cutis*, respectively. Genes are shown in different coloured boxes according to their putative functions. Genes on the outside of the maps are transcribed in the clockwise direction, and inner genes are transcribed in the counterclockwise direction. (**c**) Comparison of the gene order of the plastid genomes of *C. variabilis*, *A. protothecoides*, *P. wickerhamii*, *P. cutis*, *P. stagnora*, and *Helicosporidium* sp. Homologous genes are connected by straight lines as shown in the figure. Most of the photosynthesis-related genes (green) are absent in the non-photosynthetic lineages.

We sequenced the complete plastid and the draft nuclear genomes of two *Prototheca* species, *P. cutis* (JCM 15793 strain) and *P. stagnora* (JCM 9641 strain). The plastid genomes comprised 51.7 kb and 48.2 kb in *P. cutis* and *P. stagnora*, respectively (Fig. 1a,b); and these genomes were smaller than that of the plastid genome of *P. wickerhamii* (55.6 kb) and larger than that of *Helicosporidium* sp. (37.5 kb) (Table 1). Both plastid genomes were composed of relatively low GC (i.e. 29.7% in *P. cutis* and 25.7% in *P. stagnora*). The plastid genome of *P. cutis* was predicted to contain 72 genes, including 40 protein-coding genes, 29 tRNAs and 3 rRNAs; and its gene composition was almost identical to that of *P. wickerhamii* (Supplemental Table 1). In contrast, the *P. stagnora* plastid genome had 56 genes, including 28 protein-coding genes, 25 tRNAs, and 3 rRNAs. Both species lacked many plastid genes required for photosynthesis (e.g., genes for photosystem complexes, RubisCO large subunit, and chlorophyll biosynthesis). Although *P. stagnora* lacked all the photosynthesis-related genes, *P. cutis* retained six genes for the ATP synthase (*atpA*, *atpB*, *atpE*, *atpF*, *atpH*, and *atpI*) of the plastid similar to *P. wickerhamii* (Fig. 1b,c).

**Table 1:** General features of the plastid and nuclear genomes of *Prototheca* spp., *Helicosporidium* sp., *Auxenochlorella protothecoides*, and *Chlorella variabilis*.

Organisms	*C. variabilis*	*A. protothecoides*	*P. wickerhamii*	*P. cutis*	*P. stagnora*	*Helicosporidium* sp.
**Plastid genomes**						
Reference	HQ914635.1	Yan *et al*.^19^	Yan *et al*.^19^	This study	This study	de Koning and Keeling^20^
Genome size (kb)	124.6	84.6	55.6	51.7	48.2	37.5
GC%	33.9	30.8	31.2	29.7	25.7	26.9
Genes	115	111	72	72	56	54
Proteins	80	77	41	40	28	26
Photosynthetic proteins*	37	37	6 (atp)	6 (atp)	0	0
tRNAs	32	31	28	29	25	25
rRNAs	3	3	3	3	3	3
Spacer (bp)	460	119	122	54	98	36
**Nuclear genomes**						
Reference	Blanc *et al*. 2012	Gao *et al*.^46^	Not available	This study	This study	Pombert *et al*.^21^
Assembly size (Mb)	46.2	22.9		20.0	16.9	12.4
GC%	67	63		60.3	71.4	61.7
Proteins	9,791	7,039		6,884	7,041	6,035
Average exon size	170	207		276.8	467.5	366
Average intron size	209	246		204.4	290.3	168
Number of exons per gene	7.3	5.7		5.4	4.0	2.3
Coding%**	18.8	36.4		49.3	67.6	41.0

*Excluding conserved genes *ycf1*, *3*, *4*, *12*, *20*. **Excluding intergenic regions, introns, and ncRNAs.

For the nuclear genomes, DNA short reads were assembled into 46 and 27 large scaffolds (>1 kb) and the total sizes were 20.0 and 16.9 Mb in *P. cutis* and *P. stagnora*, respectively. Completeness of the genome assembly was estimated using BUSCO^22^ by comparing with the whole proteins available in the eukaryote database. Both the genomes abundantly recovered core eukaryotic genes in *P. cutis* (92.4%) and *P. stagnora* (88.4%), similar to the genome sequence of *A. protothecoides* (85.2%). The putative nuclear genome sizes of *Prototheca* species were smaller than that of the photosynthetic relative *Chlorella variabilis* (46.2 Mb); however, it was slightly larger than the obligate parasite *Helicosporidium* sp. (12.4 Mb) (Table 1). In these organisms, the sizes of the plastid and nuclear genomes seem to be correlated with each other (Table 1). The nuclear genomes were predicted to encode 6,884 and 7,041 proteins in *P. cutis* and *P. stagnora*, respectively. These numbers were more than the nuclear genome of *Helicosporidium* sp. (6,035 proteins), less than that of *C. variabilis* (9,791 proteins), and comparable to that of *A. protothecoides* (7,039 proteins). Therefore, no obvious difference was observed in the number of protein-coding genes between photosynthetic and non-photosynthetic trebouxiophytes. However, gene-coding capacity displayed distinct levels among the five trebouxiophyte species; non-photosynthetic species (*P. cutis*, *P. stagnora*, and *Helicosporidium* sp.) showed higher rates (41 to 67.6%) than that of the photosynthetic relatives (36.4% for *A. protothecoides* and 18.8% for *C. variabilis*).

### Phylogenetic analyses revealed multiple losses of photosynthesis in trebouxiophytes

**Figure 2:**
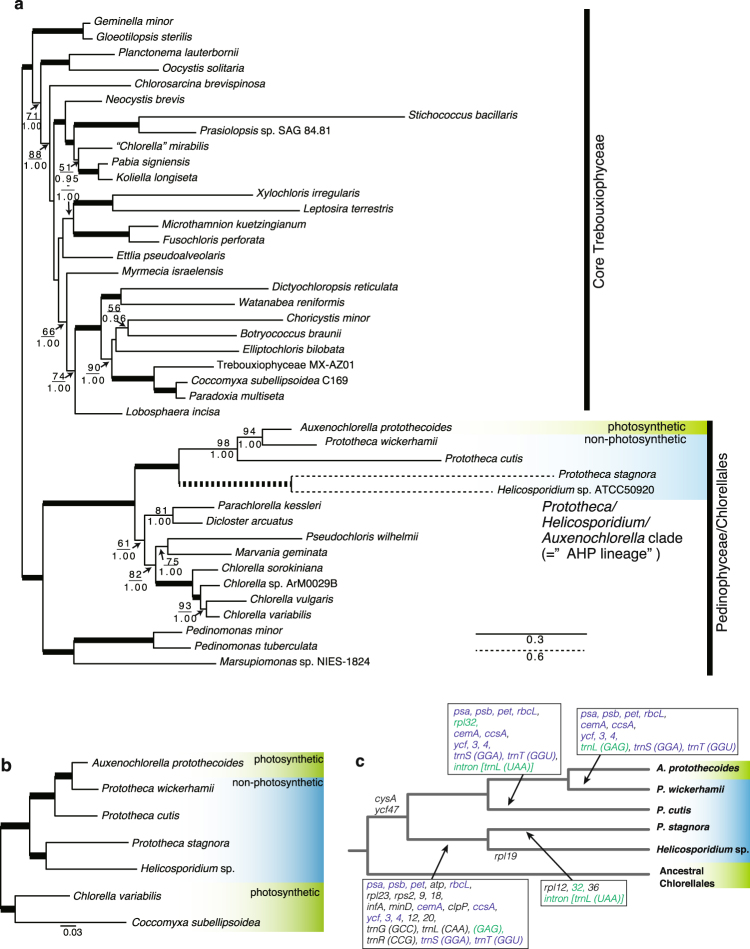
Phylogenetic tree and the evolutionary scenario of the plastid gene losses in Chlorellales. (**a**) Maximum Likelihood (ML) tree constructed using 38 plastid-encoded proteins. Bootstrap support (BP) is indicated above the lines, and Bayesian posterior probability (BPP) is indicated below the lines. BP <50 or BPP <0.95 are not shown. Bold lines represent 100% BP and 1.00 BPP. The dotted branches are shown in half-length. (**b**) ML tree of 58 nucleus-encoded proteins. All the nodes were supported with 100% BP. (**c**) Evolutionary scenario of gene losses in *P. wickerhamii*, *P. cutis*, *P. stagnora*, and *Helicosporidium* sp. Eliminated plastid genes are indicated on the tree. Genes shown in green and blue have independently disappeared two and three times in the AHP lineage. Loss of group-I intron is presented by *intron [trnL(UAA)]*.

We performed phylogenetic analyses using plastid- and nucleus-encoded proteins to reveal the evolutionary scenario pertaining to the loss of photosynthesis in trebouxiophytes. We first collected 38 plastid-encoded proteins from 42 taxa of core Trebouxiophyceae, Chlorellales, and Pedinophyceae (Supplemental Tables 2 and 3), and constructed a maximum-likelihood (ML) tree. The tree showed that three *Prototheca* species, *Helicosporidium* sp., and *A. protothecoides* formed a monophyletic group with a robust statistical support (ML bootstrap support (BP) = 100 and Bayesian posterior probability (BPP) = 1.00) within the clade of Chlorellales (Fig. 2a). *P. wickerhamii* was closely related to *A. protothecoides*, and these two were found to be sister taxa to *P. cutis*. Monophyly of *P. stagnora* and *Helicosporidium* sp. was strongly supported (BP = 100, BPP = 1.00); and they were separated from the other three taxa at the basal position. Although these relationships were well resolved, the branch lengths of *P. cutis*, *P. stagnora*, and *Helicosporidium* sp. were much longer than the others. To assess the possibility of a long-branch attraction artefact, we also constructed a phylogenetic tree using 58 nucleus-encoded proteins of *Prototheca*, *A. protothecoides*, *Helicosporidium* sp., and two photosynthetic trebouxiophytes, *C. variabilis* and *Coccomyxa subellipsoidea* (Fig. 2b). The phylogenetic tree for the nucleus-encoded proteins was topologically identical to that for the plastid-encoded proteins, and each branch was strongly supported by 100% BP.

Previous studies have reported that the three trebouxiophyte genera, *Prototheca*, *Helicosporidium*, and *Auxenochlorella*, form a monophyletic group^13,19,23,24^, and are referred to as the AHP lineage^24^. Although phylogenetic relationships within the AHP lineage have remained controversial, our phylogenetic analyses depicted a more reliable relationship of the lineage; non-photosynthetic trebouxiophytes did not show monophyly, because the photosynthetic *A. protothecoides* branched within the AHP clade. This suggests that the loss of photosynthesis has occurred in *Prototheca* and *Helicosporidium* at least three times independently in *P. wickerhamii*, *P. cutis*, and the lineage of *P. stagnora* and *Helicosporidium*. Additionally, our phylogenetic analyses also proved that the three species of *Prototheca* are either poly- or paraphyletic, suggesting that the genus *Prototheca* will require emendation in the future.

### Convergent reductive evolution of non-photosynthetic plastid genomes

The plastid genomes of *P. wickerhamii*, *P. cutis*, *P. stagnora*, and *Helicosporidium* sp. lacked 36, 37, 50, and 52 protein-coding genes compared to the photosynthetic relative *A. protothecoides* (Fig. 2c and Supplemental Table 1). The same set of 36 genes related to photosystem I and II complexes (*psa* and *psb*), cytochrome (*pet*), chlorophyll biosynthesis (*chl*), RubisCO large subunit (*rbcL*) and others (*cemA*, *ccsA*, *ycf*3, *ycf*4, and *ycf*12) was absent in all the four plastid genomes, whereas these genes were postulated to have been independently lost in each lineage based on the phylogenetic relationships. Additionally, 12 genes for ATP synthase (*atp*), translation (*rps2*, *rps9*, *rps18*, *rpl23*, and *infA*), and others (*clpP* and *ycf*20) were absent in *P. stagnora* and *Helicosporidium* sp. A few genes encoding ribosomal subunits were distinctly absent in the respective species; e.g. *rpl12* and *rpl36* were absent in *P. stagnora* and *rpl19* was absent in *Helicosporidium* sp. As these plastid genes were not found in their nuclear genomes, they were probably lost in these organisms. Two to six tRNA genes were absent in the four plastid genomes, and *trnS(GGA)* and *trnT(GGU)* genes were absent in all the genomes. Additionally, *P. cutis* and *P. stagnora* were found to lack a group-I intron that is broadly conserved in the *trnL* genes of plastid genomes^25,26^. Although gene losses independently occurred in the respective lineages of *Prototheca* and *Helicosporidium*, they affected similar sets of genes. Hence, there might be convergent reductive evolution of non-photosynthetic plastid genomes in trebouxiophytes. In terms of gene order, plastid genomes of the AHP lineage showed many syntenic regions (Fig. 1c). Interestingly, the gene order of *P. cutis* and *P. wickerhamii* was almost identical, suggesting that these two *Prototheca* species have independently eliminated the same set of plastid genes, while retaining the genome structure (Fig. 1c). In contrast, the plastid genomes of *P. stagnora* and *Helicosporidium* sp. were highly rearranged. This is probably due to the differences in the evolutionary time during which respective lineages lost their photosynthetic ability.

### ATP synthase genes in non-photosynthetic plastids

Despite being non-photosynthetic, *P. cutis* and *P. wickerhamii* retained several photosynthesis-related genes in the plastid genomes, such as the ATP synthase genes (*atpA*, *atpB*, *atpE*, *atpF*, *atpH*, and *atpI*). Transcripts of these genes have been detected by reverse transcription PCR and Northern blot analysis in *P. wickerhamii*^27^. We further confirmed that the five ATP synthase genes (*atpA*, *atpB*, *atpE*, *atpH*, and *atpI*) were transcribed in *P. cutis* at a similar level to other plastid genes (*rpL5*, *rpoB*, and *rpoC*) by reverse transcription quantitative PCR (Supplemental Fig. 1). We found that the nuclear genome of *P. cutis* carried three genes for the other subunits of the plastid ATP synthase (*atpC*, *atpD*, and *atpG*). Therefore, *P. cutis* has a full set of ATP synthase genes, which are completely absent in *P. stagnora* and *Helicosporidium* sp. To evaluate the differences in the selective pressures on the ATP synthase genes between the photosynthetic and non-photosynthetic plastid genomes, we calculated their *dN/dS* ratios. The average *dN/dS* ratio between the photosynthetic *C. variabilis* and the non-photosynthetic *P. cutis* or *P. wickerhamii* was 0.021 or 0.040, which was not significantly different from the ratio between *C. variabilis* and *A. protothecoides* (0.010), and *C. variabilis* and *C. subellipsoidea* (0.007) (*p* > 0.05, paired t-test) (Supplemental Table 4). Hence, there is no indication that the ATP synthase genes have been exposed to peculiar selective pressures during the non-photosynthetic lifestyle. Therefore, we considered that the remaining genes for ATP synthase in *Prototheca* might have some function.

Plastid ATP synthase genes were also found in the non-photosynthetic plastids of the cryptophyte *Cryptomonas paramecium*^28^ and the diatom *Nitzschia* sp.^29^. It has been proposed that ATP hydrolysis in the non-photosynthetic plastids may produce a proton gradient between the thylakoids and stroma that is involved in the protein translocation to the thylakoids by the twin arginine translocator (Tat) system^29^. Although the photosynthetic relative *A. protothecoides* has a candidate gene for the plastid TatC protein (XP_011401675), no genes for the Tat system were found in the plastid and nuclear genome of *Prototheca* by our BLAST searches. These facts implied that the ATP synthase of the *Prototheca* plastid might have some unknown functions that is not related to the thylakoid Tat system; and this function is not indispensable in *Prototheca*, because *P. stagnora* completely lacks all genes required for the plastid ATP synthase.

### Loss of nucleus-encoded plastid-targeted proteins

**Figure 3:**
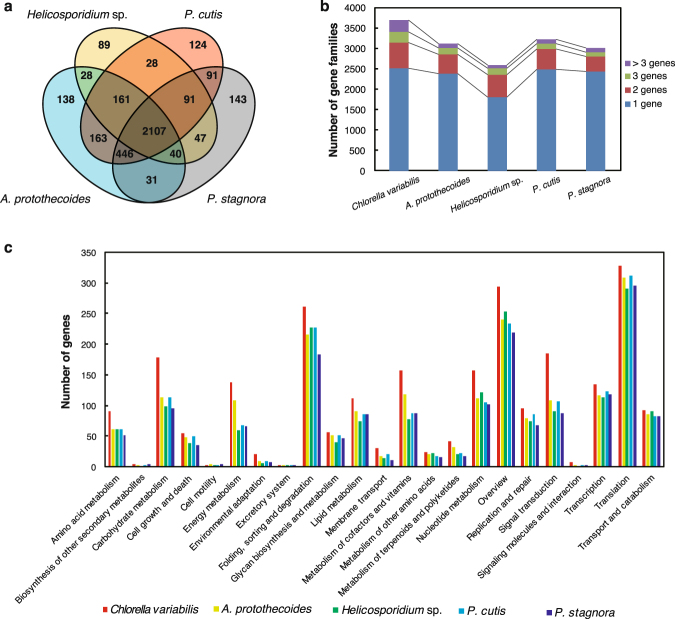
Comparison of nucleus-encoded proteins among *Prototheca*, *Auxenochlorella*, and *Helicosporidium*. (**a**) Venn diagram of shared gene families among *P. cutis*, *P*. *stagnora*, *A. protothecoides*, and *Helicosporidium* sp. (**b**) The number and size of gene families. Gene families consisting of multiple genes are shown in red, green, and purple according to their family size (two, three, and more than four). (**c**) The number of genes according to KEGG classification.

The nuclear genome sizes of *P. cutis* (20.0 Mb) and *P. stagnora* (16.9 Mb) were predicted to be smaller than that of their photosynthetic relatives, *A. protothecoides* (22.9 Mb), and *C. variabilis* (46.2 Mb) (Table 1). The nuclear genome of *Helicosporidium* sp. (12.4 Mbp) is the smallest among the available nuclear genomes of the AHP lineage, mainly because of a contraction of the gene family complexity rather than the loss of genes for certain functional categories^21^. To evaluate the complexity of gene families in *Prototheca*, we defined the orthologous genes in the respective nuclear genomes of *P. cutis*, *P. stagnora*, and *Helicosporidium* sp., as well as in *A. protothecoides* and *C. variabilis* using the TreeFam database^30^. We estimated 3,211 and 2,996 gene families in *P. cutis* and *P. stagnora*, respectively, which were similar to the number found in *A. protothecoides* (3,114 gene families) and smaller than that found in *C. variabilis* (3,688 gene families) (Fig. 3a,b; Supplemental Table 5). We also compared the number of genes according to the KEGG classification (Fig. 3c). There were no clear differences in the respective gene families and the functional categories among *Prototheca* and *Auxenochlorella* species, except for two categories; energy metabolism, and metabolism of cofactors and vitamins that had obvious connection to photosynthesis. Therefore, substantial gene loss and gain for certain functional categories other than that for photosynthesis probably did not occur during the shift from photosynthetic to teh heterotrophic lifestyle. However, *Helicosporidium* sp. carried more reduced gene families (2,591 gene families) compared to the others. Although *Prototheca* species are mainly free-living, *Helicosporidium* is the obligate parasite of insects. Therefore, it is considered that further genome reduction has to be related to the increased dependence on the host^21^.

**Figure 4:**
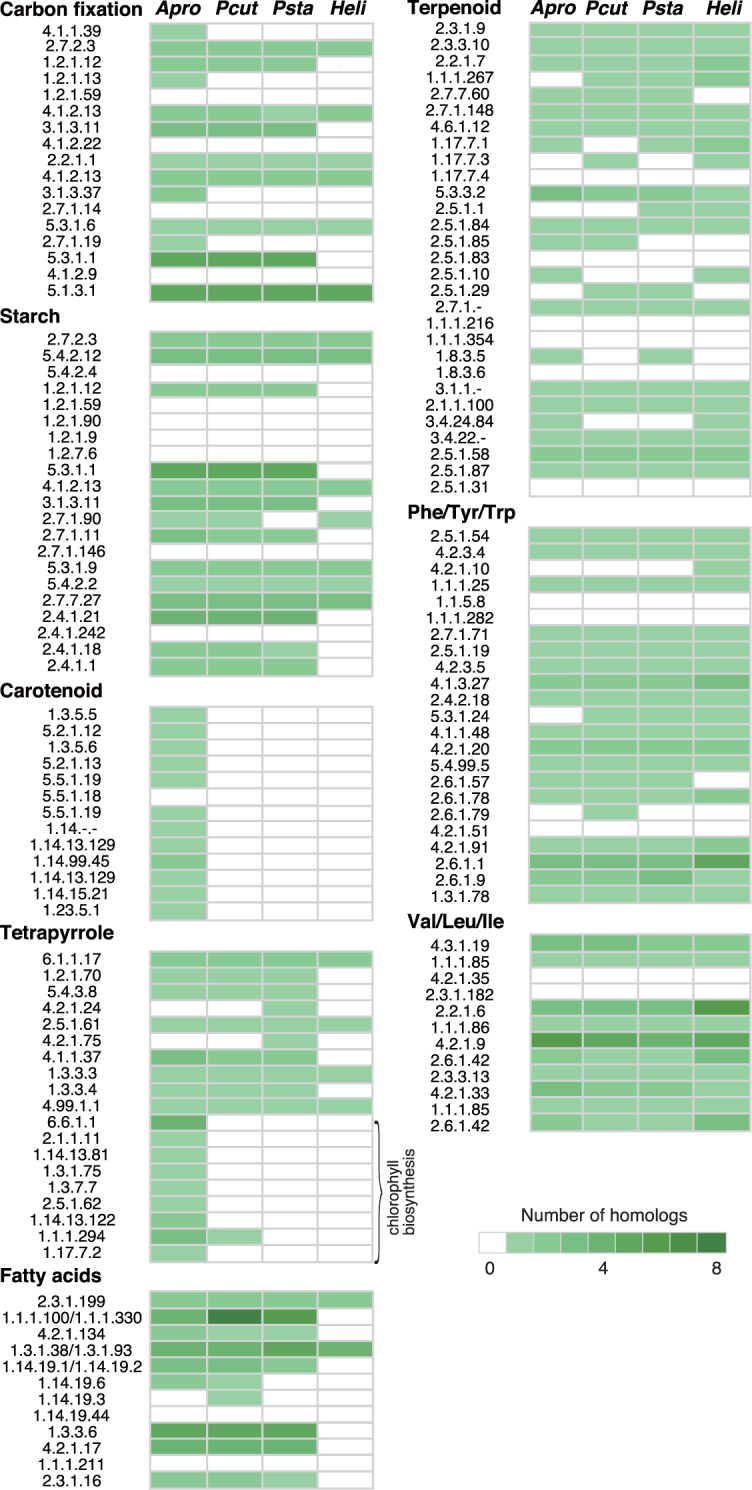
Gene contents related to plastid biosynthesis in *Prototheca*, *Auxenochlorella*, and *Helicosporidium*. Genes for plastid-related proteins were categorized into eight groups according to their functions: carbon fixation, and biosynthesis of starch, carotenoid, tetrapyrrole, fatty acids, terpenoid, Phe/Tyr/Trp, and Val/Leu/Ile. Green coloured boxes indicate the presence of genes for the plastid-related proteins as shown by EC numbers. The colour gradient represents the copy number of genes.

The colourless plastids of *P. wickerhamii* and *Helicosporidium* sp. were predicted to function in the biosynthesis of starch, fatty acids, tetrapyrrole, terpenoids, and amino acids based on their gene composition for plastid-targeted proteins^10,21^. We compared the nuclear gene contents related to metabolism in the plastids among *P. cutis*, *P. stagnora*, *Helicosporidium* sp., and *A. protothecoides* (Fig. 4). The three non-photosynthetic species depicted a similar gene content, in which genes related to carotenoid and chlorophyll biosynthesis were mostly eliminated; however, the genes for other products (e.g. starch, fatty acids, tetrapyrrole, and terpenoids) were retained. Nuclear genes for photosystems, including light-harvesting complexes were not found in the non-photosynthetic species. Therefore, elimination of the genes related to certain biological processes of the plastid has occurred concurrently in both the plastid and nuclear genomes. Exceptionally, *P. cutis* and *Helicosporidium* sp. possess a putative gene for chlorophyll *b* reductase (EC: 1.1.1.294) (Fig. 4), which converts chlorophyll *a* to *b*. However, the *dN*/*dS* ratios of this gene in *P. cutis* (0.051) and *Helicosporidium* sp. (0.050) were clearly higher than that in their photosynthetic relatives, *A. protothecoides* (0.0065) and *C*. *subellipsoidea* (0.0075); the *dN*/*dS* ratios were calculated against the gene of *C. variabilis*. Moreover, the C-terminal domain of the chlorophyll *b* reductase was truncated in *Helicosporidium*. Therefore, chlorophyll *b* reductase genes of the non-photosynthetic species would be under the process of gene disruption.

**Figure 5:**
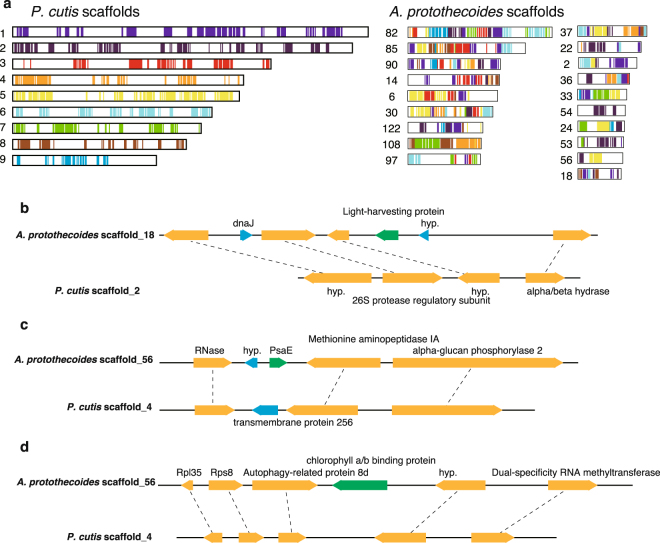
Synteny analysis of the nuclear genomes between *P*. *cutis* and *A*. *protothecoides*. (**a**) Syntenic regions between *P. cutis* and *A. protothecoides* nuclear genomes are indicated by coloured lines. Numbers beside the scheme represent a scaffold number. (**b**–**d**) Syntenic regions including photosynthesis-related genes (green) in *A*. *protothecoides*. Homologous genes (orange) between *P. cutis* and *A. protothecoides* are connected by dotted lines.

Comparison of the syntenic regions of the plastid genomes of *P. cutis*, *P. wickerhamii*, and *A. protothecoides* revealed that the colourless plastid genomes eliminated the photosynthesis-related genes, while maintaining the gene order, and the remarkable footprints of the missing genes (i.e. truncated pseudogenes) were not found in the syntenic regions (Fig. 1c)^19^. As described above, the photosynthesis-related nuclear genes have also been lost in *Prototheca* and *Helicosporidium* sp.^21^. To investigate the process of the nuclear gene reduction, comparative analyses of the syntenic regions were performed among *P. cutis*, *P. stagnora*, and *A. protothecoides*. Although the nuclear genomes represented a highly recombinant structure compared to the plastid genomes, a total of 165 syntenic blocks, including 11.9 genes on average was detected between *P. cutis* and *A. protothecoides* (Fig. 5a). *P. stagnora* and *A. protothecoides* shared 160 syntenic blocks with an average of 6.8 genes, and *P. cutis* and *P. stagnora* exhibited 275 syntenic blocks with an average of 5.8 genes (Supplemental Fig. 2). We identified three genes for the photosynthesis-related proteins, light-harvesting protein, PsaE, and chlorophyll *a*/*b* binding protein within the syntenic regions (Fig. 5b–d). The junction flanking the *psaE* gene was substituted by the gene encoding a transmembrane protein in *P. cutis* (Fig. 5c). The junctions for the other two genes were shortened and did not encode any proteins (Fig. 5b,d). These findings suggest that parts of the photosynthesis-related nuclear genes in *P. cutis* were omitted from the chromosomes without gene rearrangement similar to the plastid genome during the shift from the photosynthetic to the heterotrophic lifestyle.

## Conclusion

In this study, we report the plastid and nuclear genomes of two *Prototheca* species, *P. cutis* and *P. stagnora*. Our analyses suggest that multiple independent losses of photosynthesis have occurred in the non-photosynthetic trebouxiophytes, which have convergently lost a similar set of genes related to photosynthesis in the plastid and nuclear genomes. Such frequent losses of photosynthesis could possibly imply that some other mixotrophic relative of *Prototheca* (e.g. some species in genera *Chlorella* and *Auxenochlorella*) may eventually give up photosynthesis in future. Long-term monitoring of genome modification in *Auxenochlorella* under heterotrophic conditions will be an effective approach to investigate the possibility of an irreversible shift from mixotrophy to obligate heterotrophy.

## Materials and Methods

### Culture and DNA extraction

*P. cutis* JCM 15793 and *P. stagnora* JCM 9641 were obtained from the Japan Collection of Microorganisms, RIKEN BioResource Center (RIKEN BRC-JCM), Japan. *P. cutis* was cultured in 250 mL of YM broth (1% glucose, 0.5% peptone, 0.3% yeast extract, 0.3% malt extract, Difco) for 3 days at 30 °C under constant shaking (150 rpm), and the cells were collected by centrifugation. *P. stagnora* was cultured on YM agar at 25 °C for 10 days, followed by collecting the cells by scraping. The cell mass was freeze-dried, and ground in a mortar. Total DNA was extracted using phenol/chloroform/isoamyl alcohol, precipitated by adding 2-propanol, and then spooled out with a sterile glass rod. The crude DNA was dissolved in G2 Buffer (Qiagen, Cat. No. 1014636), and purified using a Genomic-tip 100/G (Qiagen, Cat. No. 10243) according to the manufacturer’s instruction. The DNA was further cleaned using PowerClean Pro DNA Clean-Up Kit (MO Bio Laboratories, Cat. No. 12997-50) and used for the library preparation for subsequent sequencing.

### DNA sequencing and assembly

A paired-end library with approximate insert size of 240 bp was prepared using TruSeq DNA PCR-free Library Preparation Kit (Illumina, Cat. No. FC-121-3001) according to the manufacturer’s protocol. A mate pair library with approximate insert size of 3 kbp was also prepared using Nextera Mate Pair Sample Preparation Kit (Illumina, Cat. No. FC-132-1001) with some modifications^31^. Whole genome sequencing was performed using the Illumina HiSeq. 2500 platform to generate 151-base paired-end reads. The mate pair reads were processed with NextClip v.0.8^32^ to trim the adapter sequences. ALLPATHS-LG v.52488^33^ was used to assemble both paired-end and mate pair reads into scaffolds with default parameters. The number of reads used for the *de novo* genome assemblies were 35,146,956 paired-end reads (5.3 Gb) and 11,863,706 mate pair reads (1.2 Gb) for *P. cutis*; and 63,282,152 paired-end reads (9.6 Gb) and 4,759,250 mate pair reads (5.0 Gb) for *P. stagnora*. The coverage of the paired-end reads of *P. cutis* and *P. stagnora* were approximately 265x and 568x, respectively. The N50 values of *P. cutis* and *P. stagnora* were 1.4 Mbp and 1.1 Mbp, respectively. For the reconstruction of plastid genomes, 667,790 paired-end reads (101 Mb) and 444,892 mate pair reads (44 Mb) of *P. cutis*, and 569,540 paired-end reads (86 Mb) and 366,460 mate pair reads (38 Mb) of *P. stagnora* were randomly sampled and assembled using ALLPATHS-LG with default parameters. Plastid genome sequences were identified using BLAST against the chloroplast genome sequence of *P. wickerhamii* (accession no. KJ001761).

### Gene annotation

For the annotation of plastid genomes, we initially identified the plastid genes using GeneMarkS^34^, and annotated them using BLASTx^35^. tRNAscan-SE^36^ and RNAmmer^37^ were used to predict tRNA and rRNA, respectively. All the plastid genes were manually curated on the Artemis genome browser^38^. In the case of nuclear genomes, the coding regions were predicted by MAKER annotation pipeline v.2.31.8^39^, including AUGUSTUS v.3.0.3^40^, SNAP^41^, and GeneMark-ES v.4.21^42^, wherein AUGUSTUS and SNAP were trained on *A. protothecoides* (https://www.ncbi.nlm.nih.gov/assembly/GCF_000733215.1) and *C. variabilis* (https://www.ncbi.nlm.nih.gov/assembly/GCF_000147415.1/). To estimate assembly completeness, we performed BUSCO analysis^22^ with the eukaryote dataset using the protein sequences. The estimated completeness of *P. cutis* and *P. stagnora* were 92.1% (S: 91.7%, D: 0.7%, F: 4.0%, and M: 3.6%) and 88.4% (S: 87.1%, D: 1.3%, F: 6.3%, and M: 5.3%), respectively. Functional gene annotation was performed according to the sequence homology in the Kyoto Encyclopedia of Genes and Genomes (KEGG) database^43^ using the KEGG Automatic Annotation Server (KAAS) with BBH method^44^. Conserved syntenic regions between the two nuclear genomes of *P. stagnora*, *P. cutis*, and *A. protothecoides* were searched using the CHROnicle program of SynChro (January 2015)^45^. For this analysis, we applied 7, 9, and 19 long scaffolds (>300 kb) of *P. stagnora*, *P. cutis*, and *A. protothecoides*, respectively. Syntenic blocks, including more than two orthologous genes, were identified using reciprocal BLAST hits with a similarity threshold of 40% and a length ratio of 1.3.

### Classification of gene families

Annotated nuclear genes of *C. variabilis*, *A. protothecoides*^46^, *Helicosporidium* sp.^21^, *P. cutis*, and *P. stagnora* were classified into known gene families using TreeFam 9^30^ with an E-value cut-off of 1E-5. Plastid-related proteins were identified using PRIAM (March 2015)^47^ with an E-value cut-off of 1E-10.

### Phylogenetic analyses

We performed the phylogenetic analysis using 38 highly conserved plastid encoding proteins (Supplemental Table 2), equivalent to 6,467 amino acids, representing 42 taxa (Supplemental Table 3). Organisms belonging to the core Trebouxiophyceae^48^ were used as an outgroup. The sequences were aligned using MAFFT 7.164b with the L-INS-i option^49^, and poorly aligned regions were manually eliminated using MEGA 6.0^50^. Model test was carried out by IQ-TREE multicore v.1.3.2^51^ and maximum likelihood (ML) analyses were performed with the options LG + GAMMA + I + F using RAxML v.8.1.21^52^. Statistical support was evaluated with the nonparametric bootstrap test using 200 replications. Bayesian analyses were performed using MrBayes v3.2.6^53^ with the same substitutional model. Bayesian inference consisted of 2,000,000 generations with sampling at every 1,000 generations using the four Metropolis-coupled Markov chain Monte Carlo (MCMCMC) simulations. Two separate runs were performed, and the convergence was assessed by the average standard deviation of split frequencies (ASDSF) falling below 0.01. Bayesian posterior probabilities (BPP) were calculated from the majority rule consensus of the trees sampled after the initial 500 burn-in trees.

We also performed phylogenetic analyses using the nucleus-encoded proteins of 7 taxa (*P. cutis*, *P. stagnora*, *P. wickerhamii*, *Helicosporidium* sp., *A. protothecoides*, *C. variabilis*, and *C. subellipsoidea*). Orthologous sequences among these taxa were searched using the reciprocal best-hit analyses with the cut-off: similarity >70% and HSP coverage >50%. A total of 58 proteins, which were shared by at least six taxa, were used for the analyses (Supplemental Table 6). ML analyses were performed using the same method with the plastid-encoded proteins.

### Nucleotide substitution rates of synonymous (*dS*) and nonsynonymous (*dN*) sites

The *dN*/*dS* ratios of the plastid-encoded ATP synthase genes and chlorophyll *b* reductase genes were calculated for *P. cutis*, *P. wickerhamii*, *A. protothecoides*, *C. variabilis*, and *C. subellipsoidea*. Amino acid sequences were aligned using MAFFT 7.164b with the L-INS-i option. The aligned sequences were converted to nucleotide sequences using PAL2NAL v.14^54^. Pairwise *dN*/*dS* ratios among *C. variabilis* and the others were calculated using the codeml program of the PAML package v.4.8^55^.

### Data deposition

The plastid and nuclear genome sequences of *P. cutis* JCM 15793 and *P. stagnora* JCM 9641 were deposited in DDBJ/GenBank/ENA under accession numbers AP018373 (*P. cutis* plastid), AP018372 (*P. stagnora* plastid), BCIH01000000 (*P. cutis* nuclear), and BCJY01000000 (*P. stagnora* nuclear).

## Electronic supplementary material

Supplemetary Methods and Figures

Supplementary Tables
