# Supplementary material for: Multiple losses of photosynthesis and convergent reductive genome evolution in the colourless green algae Prototheca
Source: Sci Rep. 2018 Jan 17;8:940. doi: 10.1038/s41598-017-18378-8 (PMC5772498; doi:10.1038/s41598-017-18378-8)
Supplement: Supplementary file 1 — Supplemetary Methods and Figures [file 41598_2017_18378_MOESM1_ESM.pdf]

# Supplementary Information

## Multiple losses of photosynthesis and convergent reductive genome evolution in the colourless green algae *Prototheca*

Shigekatsu Suzuki[1, 2], Rikiya Endoh[3], Ri-ichiroh Manabe[4], Moriya Ohkuma[3], and Yoshihisa Hirakawa[5*]

[1]Graduate School of Life and Environmental Sciences, University of Tsukuba, Ibaraki, Japan

[2]Center for Environmental Biology and Ecosystem Studies, National Institute of Environmental Studies, Ibaraki, Japan

[3]Japan Collection of Microorganisms, RIKEN BioResource Center, Ibaraki, Japan

[4] Division of Genomic Technologies, RIKEN Center for Life Science Technologies

[5]Faculty of Life and Environmental Sciences, University of Tsukuba, Ibaraki, Japan

* Corresponding author: Yoshihisa Hirakawa, Faculty of Life and Environmental Sciences, University of Tsukuba, Japan, Email: hirakawa.yoshi.fp@u.tsukuba.ac.jp

**Supplementary Methods**

**Reverse transcription quantitative PCR (RT-qPCR) for ATP synthase genes**

Total RNA was extracted from *Prototheca cutis* JCM 15793 by using TRIzol reagent (Invitrogen). The cells were collected from 50 mL culture in YM broth, and then disrupted in 1 mL TRIzol reagent with 0.5 g silica beads (0.2 mm) by Beads Crusher μT-01 (Taitec) at 4,600 r/min for 10 sec. RNA was purified from the cell lysate according to the manufacturer's protocol. The extracted RNA was treated with DNase I (Invitrogen, Cat. No. 18068015) to remove DNA contamination. Reverse transcription was performed with 5 μg DNase-treated RNA in total 50 μL reaction mixture of ReverTra Ace qPCR RT Kit (Toyobo, Cat. No. FSQ-101) according to the manufacturer's protocol; we also prepared a reaction mixture without transcriptase as a negative control (RT minus). Primers for quantification of ATP synthase genes (*atpA*, *atpB*, *atpE*, *atpH*, and *atpI*) and other plastid genes (*rpL5*, *rpoB*, and *rpoC1*) were designed using the Primer3Plus online software: TCTTTCGCTCCCGGGATTATTG and ATTAATTCACGCTGCCCTCGAC for *atpA*, TCGGTTTTTGCTGGAGTAGGAG and TCGTGCACCTGGAGATTCATTC for *atpB*, TCCTTCCAACTACCACAGGA and GCTTTATAATAGCAAATCCC for *atpE*, AGCGTCATTGCTGCAGGTATTG and AATGCATATCCCGCAGCTGTAC for *atpH*, ACCCCTTTCCTTAAGCTTTCGC and TGGGACTGGAAGAAACAATGGG for *atpI*, TTGTTGTGCATCGTGGTCTTGG and TCGAATAGCACCATGTTGTCCTG for *rpL5*, GGGCGTAAATACACAACAAGCG and ATCCAGCGTTTTCTCCTTCTGG for *rpoB*, AATATCCTTGCTCCCGCTTCTG and GACGCTGGGGCAGTTAAATAAAAAC for *rpoC1*. We failed to design optimal primers for *atpF*, because of its extremely high AT content (84%). RT-qPCR was performed using a Thermal Cycler Dice Real Time System II (Takara) under the following conditions: 1 μL of RT reaction mixture, 0.4 μM of each primer, 12.5 μL of SYBR Premix Ex Taq II (Takara, Cat. No. RR820A), and DNase/RNase-free water up to 25 μL. The cycling conditions comprised 3 min of denaturation at 95 °C, followed by 40 cycles of 10 s at 95 °C and 30 s at 60 °C, and a melting curve program for detection of non- specific products. To evaluate transcript abundance of each gene, CT values were compared between samples with and without reverse transcriptase (RT plus and RT minus) using the ΔΔCT method.

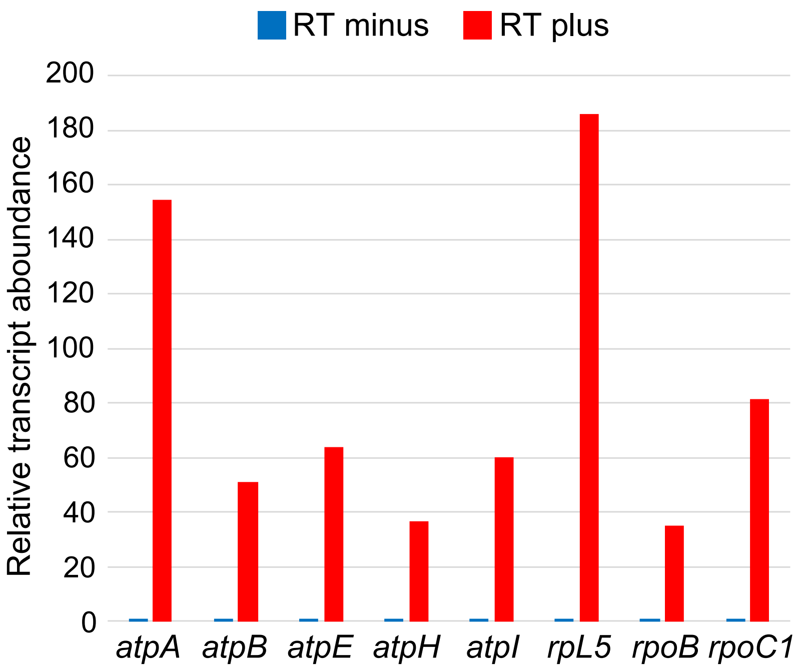


**Supplementary Fig. 1.** Relative transcript abundance of plastid ATP synthase genes (*atpA*, *atpB*, *atpE*, *atpH*, and *atpI*) and other plastid genes (*rpL5*, *rpoB*, and *rpoC1*) in *Prototheca cutis*. Quantitative PCR was performed with two templates that were reacted with and without reverse transcriptase (RT plus and RT minus) (see Supplementary methods).
The CT values of RT minus are present the amount of DNA contamination. The transcript abundance of each gene was normalized by the value for RT minus (the values of RT minus were set to 1).

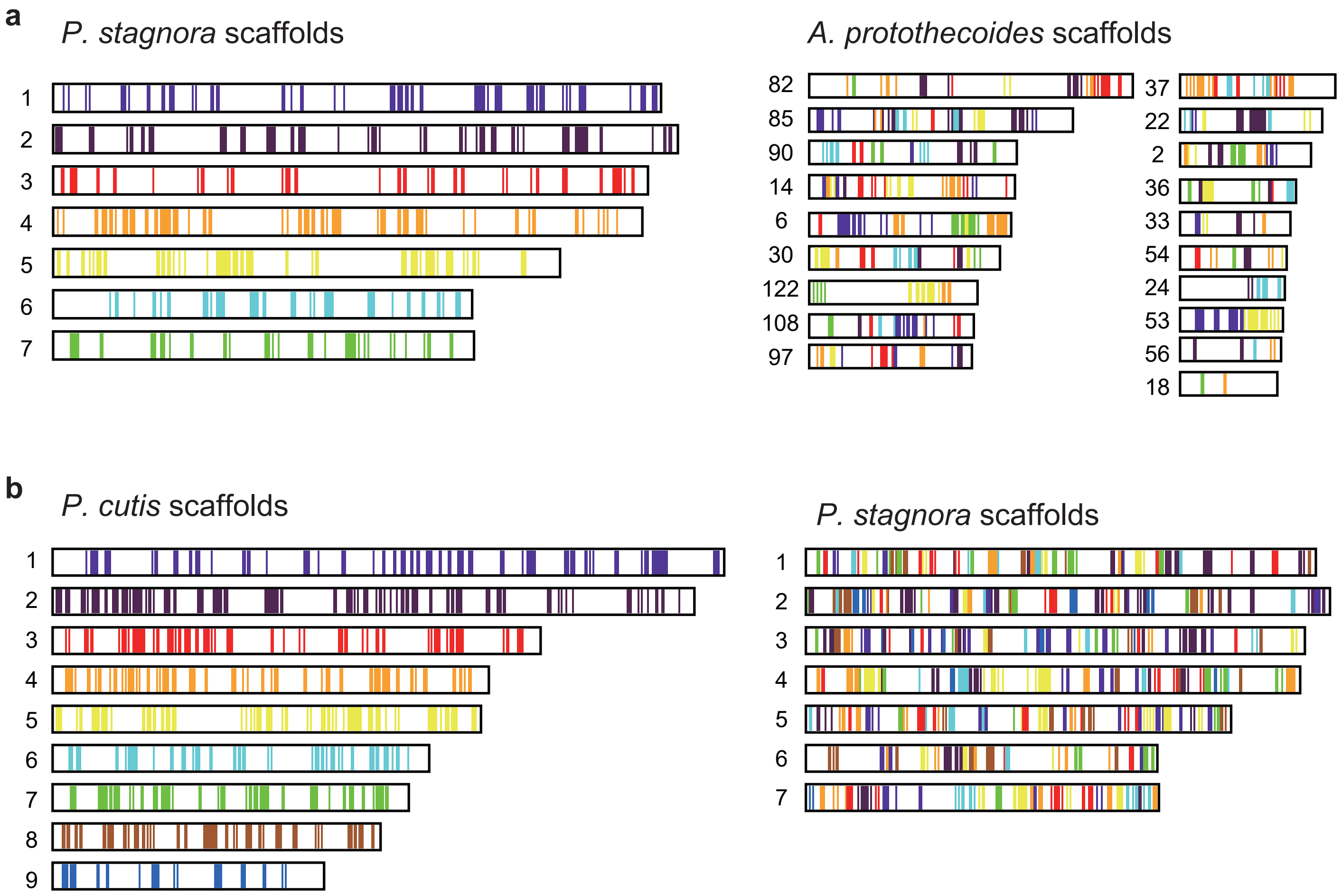


**Supplementary Fig. 2.** Synteny analysis of the nuclear genomes between *P. stagnore* and *A. protothecoides* (a) and *P. cutis* and *P. stagnora* (b). Syntenic regions are indicated by coloured lines.
*P. stagnora* and *A. protothecoides* shared 160 syntenic blocks with 6.8 genes on average, and *P. cutis* and *P. stagnora* exhibited 275 syntenic blocks with 5.8 genes on average
